# Strategies for Enhancing the Implementation of Universal Mental Health Prevention Programs in Schools: A Systematic Review

**DOI:** 10.1007/s11121-022-01434-9

**Published:** 2022-09-13

**Authors:** Rachel Baffsky, Rebecca Ivers, Patricia Cullen, Jessica Wang, Lauren McGillivray, Michelle Torok

**Affiliations:** 1 School of Population Health, UNSW Sydney, Samuels Building F25 Samuel Terry Ave, Kensington, NSW Australia; 2 Black Dog Institute, University of New South Wales, Hospital Road, Randwick, NSW Australia

**Keywords:** Prevention, Implementation science, Systematic review, Mental health, School-based interventions

## Abstract

A number of school-based mental health prevention programs have been found to be effective in research trials, but little is known about how to support implementation in real-life settings. To address this translational problem, this systematic review aims to identify effective strategies for enhancing the implementation of mental health prevention programs for children in schools. Four electronic databases were searched for empirical, peer-reviewed articles in English from January 2000 to October 2021 reporting the effects of implementation strategies for school-based universal mental health programs. Twenty-one articles were included in the narrative synthesis and assessed for quality using the Mixed Methods Appraisal Tool. Twenty-two strategies were found to be effective at improving program fidelity or adoption. The strategies with the strongest positive evidence base were those that involved monitoring and provision of feedback, engaging principals as program leaders, improving teachers’ buy-in and organising school personnel implementation meetings. We recommend school-based practitioners trial strategies with positive findings from this review as part of their continuous quality improvement. This review highlights the pressing need for large-scale, randomised controlled trials to develop and trial more robust strategies to enhance adoption, as the five implementation studies found to measure adoption used qualitative methods limited by small samples sizes and case study designs.

**Supplementary Information:**

The online version contains supplementary material available at 10.1007/s11121-022-01434-9.

## Background

Approximately 50% of mental health challenges develop before the age of 14 years (Kessler et al., 2007). Childhood mental health challenges are associated with poor health, educational and social outcomes in adulthood including mood, anxiety and externalising disorders (Mulraney et al., 2021); lower educational attainment and earnings (Copeland et al., 2021); homelessness (Grattan et al., 2022); substance use disorders; smoking; and imprisonment for violent crimes (Kellam et al., 2011). There is a need to target evidence-based prevention programs to children and young adolescents to prevent the onset of these challenges and minimise its associated risks (Arango et al., 2018).

Schools are considered the most cost-effective setting to deliver mental health prevention programs to large cohorts of children and adolescents, as a setting in which they spend a large majority of their time (Fazel et al., 2014; Williams et al., 2020). In developed countries, it is compulsory for all children to attend school and so school-based programs provide more equitable access to mental health prevention programs compared to other settings, such as communities or hospitals (Weare & Nind, 2011). Approximately 70–80% of children who utilise mental health programs do so at school (Nadeem et al., 2018). Furthermore, schools in developed countries often have existing policies that align with mental health prevention (O'Dea et al., 2021). Programs that integrate well with existing policies are more likely to be sustainable (Herlitz et al., 2020).

In developed countries, schools often take a multi-tiered approach to allocating resources for mental health programs in schools (Berger, 2019). Universal programs (tier 1) are delivered to all students regardless of risk; selected programs (tier 2) are delivered to some students at risk of mental health challenges; indicated programs (tier 3) are delivered to students displaying mental health challenges (Cook et al., 2015).

Universal approaches to school-based mental health prevention programs have advantages over targeted approaches; they can reach and benefit all children regardless of risk to increase protective factors, and, accordingly, are less likely to be stigmatising (Greenberg et al., 2017; Werner-Seidler et al., 2017). There is evidence that school-based universal mental health prevention programs can improve emotional outcomes (including resilience, self-efficacy and coping skills) (Fenwick-Smith et al., 2018) and reduce behavioural problems (e.g. office disciplinary referrals) (Bradshaw et al., 2012) and anxiety, mood and behavioural disorders in primary school-aged children (Sutan et al., 2018). Whilst several of these evidence-based programs (such as PAX Good Behaviour Game (Johansson et al., 2020) and the Promoting Alternative Thinking Strategies (Kusche, 2012)) are available for implementation, little is known about the supports required to optimise implementation which will lead to better effectiveness outcomes for children and adolescents (Proctor et al., 2009).

There is a growing body of mixed methods research that has identified key barriers–and to a lesser extent–facilitators of mental health program implementation in the school setting. General barriers include lack of training and administrative support, time constraints, staff turnover (Jack et al., 2020; Herlitz et al., 2020), academia taking priority over prevention programs (Coombes et al., 2016) and staff burnout (Domitrovich et al., 2015). Barriers more unique to youth engaging with mental health–focused programs include stigma and embarrassment (Colizzi et al., 2020; Gulliver et al., 2010), concerns about confidentiality and lack of awareness of existing programs (Gulliver et al., 2010; Kahl et al., 2020). A key facilitator of school-based mental health prevention programs is having a pool of non-mental health professionals, such as teachers, available to implement the program (Owens et al., 2014). Other facilitators include having an in-built professional network for peer discussion, regular consultation available to support delivery, monthly team meetings to resolve common implementation challenges (Beames et al., 2021; Wu et al., 2019), sufficient funding (Langley et al., 2010), support from principals and leadership team (Beames et al., 2021; Herlitz et al., 2020) and teachers receiving positive feedback of program outcomes (Dijkman et al., 2017).

Implementation science is increasingly being used in the development and testing of strategies to improve the implementation outcomes of evidence-based programs in schools (Proctor et al., 2013). An implementation outcome is the success of a purposeful action to implement a new evidence-based program as a routine school practice (Proctor et al., 2011). It is distinct from an effectiveness outcome which is a change in health as a result of the evidence-based program (Proctor et al., 2011). Proctor et al. (2011) identified eight implementation outcomes considered to enhance the effect an evidence-based program has on health outcomes: fidelity, adoption, reach, appropriateness, acceptability, feasibility, sustainability and costs. Whilst research has indicated that better implementation outcomes lead to better student mental health outcomes (Durlak & DuPre, 2008; Proctor et al., 2009), implementation strategies have typically been trialled unsystematically or under different nomenclatures (e.g. the Effective Practice and Organization of Care (EPOC) taxonomy (Effective Practice and Organization of Care, 2015) or the Expert Recommendations for Implementing Change (ERIC) taxonomy (Powell et al., 2015)). This has limited current understandings of which implementation strategies enhance which implementation outcomes for mental health prevention programs delivered in school settings.

Implementation strategies are defined as the methods used to improve the implementation outcomes of evidence-based programs (Proctor et al., 2013). To guide, improve, and standardise school implementation research, efforts have been made to create a common nomenclature of strategies. Cook et al. (2019) developed the School Implementing Strategies, Translating ERIC resources (SISTER) framework in 2019 (Cook et al., 2019). SISTER lists and defines 75 implementation strategies considered by educational experts to be useful for improving implementation of school-based programs (Cook et al., 2019). So far, the SISTER-defined strategies have been predominantly applied and tested with physical health prevention programs. There is low-medium quality evidence that strategies such as audit (monitor) and provide feedback, executive support, recognition (of implementers’ efforts), email reminders, program champions, educational materials and educational meetings enhanced implementation of physical health programs when combined with other strategies such as marketing in schools, professional networking and provision of equipment (Sutherland et al., 2020, 2021; Wolfenden et al., 2017). The existing evidence base on implementation strategies in schools has mostly focused on physical health prevention programs rather than mental health prevention programs. Furthermore, existing systematic reviews testing implementation strategies for school-based social, emotional and behavioural programs have used only single-case experimental design studies (Merle et al., 2022; Noell et al., 2014; Solomon et al., 2012; Stormont et al., 2015).

To better understand what strategies work for enhancing implementation of mental health prevention programs in schools, the overarching aim of this systematic review is to examine all types of empirical research on strategies used to implement mental health promotion or prevention programs in school settings. The review focuses on two research questions:

1. Which strategies work (and do not work) to enhance the implementation of mental health promotion or prevention programs in schools?
2. What implementation outcomes are assessed?

## Method

This systematic review was registered in PROSPERO (CRD42020208358).

### Search Strategy and Selection Criteria

Consistent with the Preferred Reporting Items for Systematic Reviews and Meta-Analyses (PRISMA) Statement, the search strategy included two steps. First, a comprehensive literature search was performed on October 6, 2021, using the following electronic bibliographic databases: PubMed (January 2000–October 2021); ERIC (January 2000–October 2021); APA PsycInfo (January 2000–October 2021); CINAHL (January 2000–October 2021). The search included a combination of four key blocks of terms related to (a) mental health and mental well-being, (b) school, (c) intervention and program and (d) implementation strategies, which were entered in the appropriate search fields (e.g. title, abstract, key words/text words and subject headings) and adapted to meet the requirements of each database. For the full search strategies, see Supplementary File 1. Second, the reference lists of relevant papers were examined to identify any additional relevant papers. The completed PRISMA checklist can be found in Supplementary File 2.

The inclusion criteria for papers were as follows: (a) population–teachers and school support staff (including principals); (b) intervention–implementation strategy designed to enhance the implementation outcomes of evidence-based universal mental health prevention programs for children/adolescents in schools. A universal prevention program was considered evidence-based if it had demonstrated positive effects in an effectiveness or efficacy trial that used a randomised controlled or quasi-experimental design and met the following standards for study quality, either fully or partially, set out by the What Works Clearinghouse (2020): (1) group assignment (for RCTs), (2) tolerable sample attrition and (3) equivalent baseline outcomes between groups (What Works Clearinghouse, 2020). The strategy could be targeted at the teacher (provider) or school (organisational/setting) level. The strategy could be standalone or multicomponent; (c) setting–primary or secondary school context; (d) outcome–primary outcome is one of the eight implementation outcomes identified by Proctor et al. (2011) as influential in implementation research (acceptability, adoption, appropriateness, costs, feasibility, fidelity, reach and sustainability); (e) study type–randomised controlled trials (with all types of control groups), quasi-experimental designs, mixed methods studies and qualitative and observational studies; (f) publication type–peer-reviewed journal articles and books; (g) country–any country; (h) language–published in English because we did not have the resources to translate non-English language studies; (i) date–published after 2000, as implementation science in its current form emerged as a discipline in the early 2000s (Bauer et al., 2015; Eccles & Mittman, 2006). Furthermore, school-based mental health practices have changed significantly over the past 20 years, increasingly prioritising curriculum-based prevention programs that integrate social and emotional learning with academic learning (World Health Organization, 2021). We do not believe programs implemented prior to 2000 are relevant to understanding current best practice.

The exclusion criteria for papers were as follows: (a) subjects–non-human participants; (b) intervention–implementation strategy targeted selective or indicated interventions. Universal prevention programs target the whole classroom, whereas selective/indicated programs do not, and it is hypothesised that the whole-classroom setting might affect which strategies worked and which did not; (c) setting–preschool or post-secondary school context; (d) publication type–non-peer-reviewed journal articles and books, conference abstracts and proceedings, dissertations, editorials, reviews, viewpoints/perspectives, study protocols and grey literature. We excluded grey literature as it is challenging to search grey literature in a systematic and replicable manner (McClain et al., 2021), and because the quality of grey literature studies cannot be assured due to the lack of peer review (Schmucker et al., 2017).

RB and JW were the coders for assessing study inclusion. MT was responsible for resolving conflicts. Prior to coding, RB, JW and MT met for two 90-min training workshops. RB led the training and clarified the aims of the review and operationalisations of the inclusion/exclusion criteria. She also developed a codebook in Microsoft Excel listing the inclusion/exclusion criteria against which titles/full texts would be screened to ensure consistency and transparency in coding. During the training, the reviewers practiced screening together.

Covidence systematic review software was used to manage citations and conduct screening. RB and JW independently screened a random selection of 15% of titles and abstracts based on the eligibility criteria to check for accuracy. Inter-rater agreement was substantial (94%, *k* = 0.65) (Landis & Koch, 1977). Discrepancies were reviewed by MT and discussed between the three authors until consensus was reached. Given our inter-rater reliability and the reviewer time required for dual screening, we made the decision for RB to single screen the remaining title and abstracts in line with other reviews (Belur et al., 2021; Merle et al., 2022; Page et al., 2021).

RB and JW independently screened a random sub-selection of 10% of full texts to test for accuracy. Inter-rater agreement was near perfect (94%, *k* = 0.83) (Landis & Koch, 1977). The few discrepancies were resolved by discussions between the two authors. In light of the high inter-rater reliability, RB single screened the remaining full-text articles.

### Data Extraction and Synthesis

RB extracted data for all included studies using a data extraction codebook in Microsoft Excel. JW extracted data for a subset of 50% of studies to check for inter-coder agreement. Extracted information included the following:

- Study-level characteristics (such as study design, sample size, data collection methods).
- Program name (identifying the universal mental health intervention being evaluated).
- Implementation strategies; using Cook et al.’s (2019) framework, we identified the SISTER-defined strategies evaluated in each included study. RB coded the seven key characteristics of each implementation strategy according to Proctor et al.’s (2013) reporting guidelines: (a) actor (who delivered the strategy), (b) action (what was done), (c) target (who was it intended for), (d) temporality (when was it used), (e) dose (frequency/quantity) and justification (empirical, theoretical or practical rationale) (Proctor et al., 2013). Prior to coding, RB reviewed the Proctor et al. paper and five school-based articles in which it was cited (Evenhuis et al., 2021; Hooley et al., 2020; Livet et al., 2018; Moore et al., 2021; Nettlefold et al., 2021) as training for how to report SISTER strategies.
- Evidence of effect of implementation strategies on implementation outcomes.

Eligible primary outcomes were implementation outcomes measured through self-report survey, observation (for fidelity) or qualitative interview with school staff, for example fidelity, adoption, acceptability, feasibility, sustainability, reach, appropriateness and costs. No data collection timeframe/period was excluded.

Data was synthesised qualitatively (narrative summary). The narrative summary described the main characteristics and results of included studies, with a focus on information about the implementation strategies used to improve or enhance implementation of mental health promotion or prevention programs in schools.

Mixed methods analysis was used to synthesise and compare studies. We identified the number of quantitative studies that had a positive effect for each strategy and the number of qualitative studies that reported positive findings for the same strategy. Together we combined that to the number of studies found to have positive findings. In text, we describe whether the findings were predominantly quantitative/or qualitative and the implications for interpretation. We analysed the quality of different study types and discussed how study type may have affected our findings.

### Quality Assessment

RB and LM independently rated the risk of bias of each included study using the Mixed Methods Appraisal Tool (MMAT) (version 2018) which was developed to appraise the quality of systematic reviews that include qualitative, quantitative and mixed methods studies (Hong et al., 2018). Discrepancies were resolved through discussion and, if necessary, consultation with a third reviewer (MT). The MMAT has been used in similar systematic reviews (Cassar et al., 2019; Fenwick-Smith et al., 2018; Shoesmith et al., 2021).

The reviewers rated each included study based on seven quality criteria. The first two criteria asked if there were clear research questions, and if the data collection methods were sufficient to answer these research questions. The reviewers responded with ‘Yes’, ‘No’ or ‘Can’t Tell’. The subsequent five quality criteria differed depending on the type of study design (qualitative, quantitative–randomised control trial; quantitative–non-randomised; quantitative–descriptive or mixed methods). We decided to include low-quality studies and transparently discuss how their methodological flaws might have impacted results (Higgins et al., 2011), as recommended by the developers of MMAT and published users (Cassar et al., 2019; Hong et al., 2018; Shoesmith et al., 2021). This was intended to prevent a selection bias, created by excluding low-quality studies that often have smaller samples and larger effect sizes (Stone et al., 2019).

## Results

### Study Selection

**Fig. 1:**
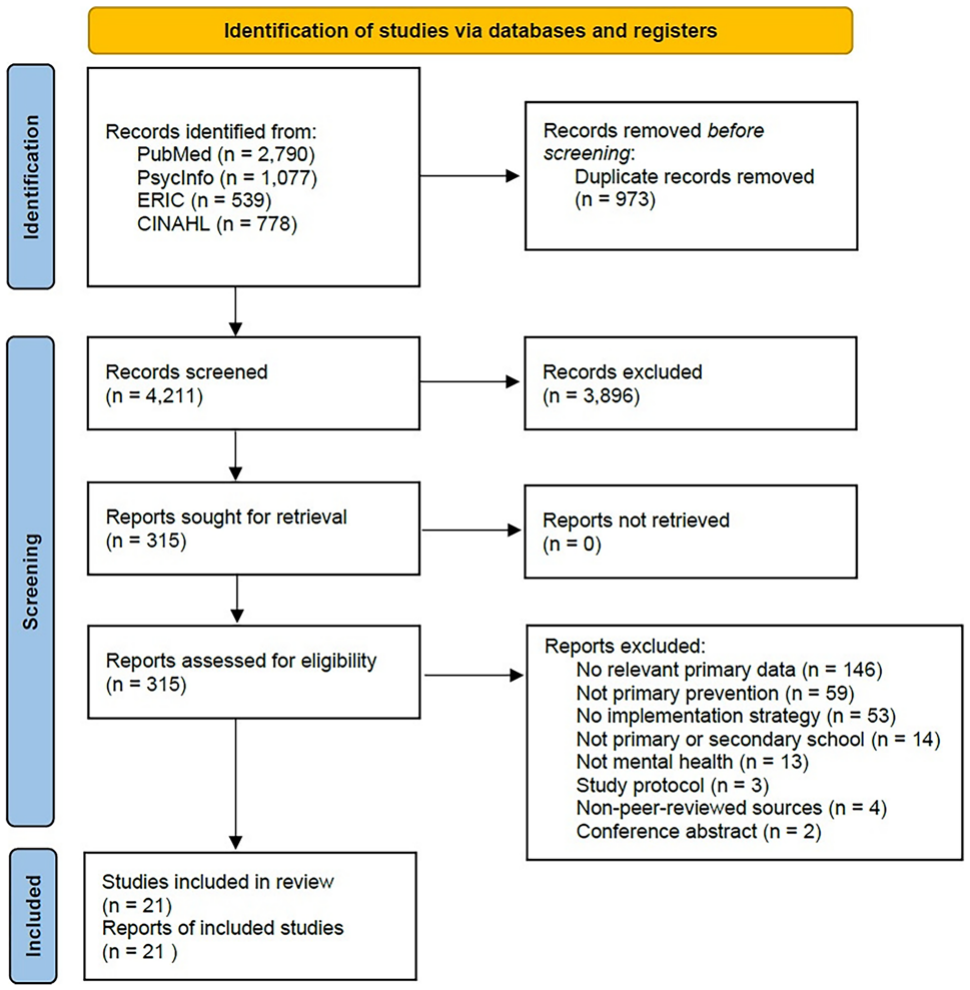
PRISMA flow diagram detailing systematic search strategy (Page et al., 2021)

Figure 1 outlines the screening process for this study. A total of 4211 articles were screened based on title and abstracts, of which 315 articles were screened by full text. Twenty-one articles were eligible for inclusion. The most common reasons for exclusion were as follows: no relevant primary data (50%), no implementation strategy (17%), not a primary prevention program (20%), not a primary or secondary school (5%) and not a mental health program (5%).

### Article Characteristics

#### Type and Quality

The country and evidence-based program in each included study is presented in Supplementary File 4. Nineteen out of 21 studies (90%) were conducted in the USA, one (5%) in Canada and one (5%) in Australia. Seven studies (33%) focused on the PAX Good Behaviour Game, and three of those also incorporated the Promoting Alternative Thinking Strategies (PATHS) program. Seven studies (33%) evaluated School-wide Positive Behavioural Interventions and Supports (SW-PBIS). The remaining seven studies were on other evidence-based programs.

Three studies (14%) used mixed methods designs (Anyon et al., 2016; Livet et al., 2018; Poduska & Kurki, 2014) and five were randomised controlled trials (RCTs) (Becker et al., 2014; Bradshaw et al., 2008, 2010; Fallon et al., 2018; Reinke et al., 2012). Seven studies used quantitative non-experimental designs including single arm (non-experimental) design with pre-post analysis (3 studies) (Cook et al., 2015; McDaniel & Bloomfield, 2020; von der Embse et al., 2019), cross-sectional study (2 studies) (Johnson et al., 2018; Pas et al., 2015), and multiple baseline design (2 studies) (Hagermoser Sanetti et al., 2018; Oliver et al., 2015). Six studies used qualitative designs (Arnold et al., 2020; Freeman et al., 2014; Hudson et al., 2020; Leadbeater et al., 2012; Lohrmann et al., 2008; Mendenhall et al., 2013).

Quality appraisal ratings can be found in Supplementary File 3. The qualitative studies were considered to be of high quality, with four out of six studies meeting all seven quality criteria (Arnold et al., 2020; Hudson et al., 2020; Leadbeater et al., 2012; Mendenhall et al., 2013). The other two qualitative studies (Freeman et al., 2014; Lohrmann et al., 2008) had not adequately based their findings on derived data and we could not tell if ‘the interpretation of results were sufficiently substantiated by the data’ and if there was ‘coherence between qualitative data sources, collection, analysis and interpretation’. The quantitative non-experimental studies were of relatively high quality with five out of seven studies meeting all seven of the quality criteria (Cook et al., 2015; Johnson et al., 2018; McDaniel & Bloomfield, 2020; Oliver et al., 2015; Pas et al., 2015). The other two quantitative non-experimental studies met six of the quality criteria; however, there was not enough detail about the demographics of the target population to determine if the sample was representative (Hagermoser Sanetti et al., 2018; von der Embse et al., 2019).

No RCTs met all seven quality criteria. None of the RCTs had outcome assessors blinded to the intervention. This was not concerning as it is not feasible or common for outcome assessors to be blinded to an intervention in educational research. Three of the five RCTs met six out of seven quality criteria (Becker et al., 2014; Bradshaw et al., 2008, 2010). It was unclear whether two out of five RCTs had an adequate randomisation process (Fallon et al., 2018; Reinke et al., 2012). One of these studies also did not have clear research questions, meeting three out of seven quality criteria, and was deemed to be low quality (Reinke et al., 2012).

Only one out of three mixed methods studies met all seventeen quality criteria (Anyon et al., 2016). The second mixed methods study did not have a representative sample but was still deemed to be of acceptable quality (Livet et al., 2018). The last mixed methods study only met one out of seventeen quality criteria and was considered to be low quality (Poduska & Kurki, 2014).

### Implementation Strategies Tested

A full overview of the implementation strategies tested in each evaluation is presented in Supplementary File 4. Each strategy has been defined using Cook et al.’s (2019) SISTER framework and reported according to Proctor et al.’s (2013) guidelines.

Twenty-four out of 75 SISTER-defined implementation strategies were tested. Supplementary File 5 tabulates the number of studies that tested each strategy in order of most to least tested strategy. All studies tested multicomponent strategies (100%).

The most commonly tested strategies (combined with other strategies) were ‘conduct ongoing training’ and ‘provide ongoing consultation/coaching’, which were each identified in 14 (67%) studies. Other commonly tested strategies were ‘audit and provide feedback’ (six studies); ‘provide local technical assistance’ (four studies); ‘inform local opinion leaders’, ‘improve implementers’ buy-in’ and ‘use train-the-trainer strategies’ (3 studies each); ‘increase demand and expectations for implementation’, ‘organize school personnel implementation team meetings’, ‘identify and prepare champions’, ‘distribute educational materials’ and ‘conduct local needs assessment’ (2 studies each). The remaining 12 strategies outlined in Supplementary File 5 were evaluated in one study.

### Effectiveness of Strategies

#### Conduct Ongoing Training

Nine of the 14 (64%) studies testing ‘ongoing training’ had positive findings. Six quantitative studies found ‘ongoing training’ had a positive effect on fidelity, where acceptable fidelity (i.e. 80% or more of the program being delivered as intended) was achieved (Hagermoser Sanetti et al., 2018; Horner et al., 2004; Sterling-Turner et al., 2002). On the other hand, five quantitative/mixed methods studies found no effect of ‘ongoing training’ on fidelity. One of these studies with null findings was rated as low quality (Poduska & Kurki, 2014). Three qualitative studies found ‘ongoing training’ to be positively associated with program adoption (defined as the school staff making the initial decision/early efforts to use an evidence-based program as routine practice) (Proctor et al., 2011).

#### Provide Ongoing Consultation/Coaching

Eight out of 14 (57%) studies assessing ‘provide ongoing consultation/coaching’ reported positive findings. Six quantitative/mixed methods studies found a positive effect of ‘provide ongoing consultation/coaching’ on fidelity. Two qualitative studies reported positive findings for program adoption. Six quantitative/mixed studies found no effect of ‘provide ongoing consultation/coaching’ on fidelity.

#### Provide Local Technical Assistance

Local technical assistance refers to the use of school staff to resolve implementation challenges (Cook et al., 2019). Three of four (75%) studies testing ‘provide local technical assistance’ found positive findings. One qualitative study reported positive findings for program adoption and two quantitative studies found a positive effect on fidelity. The study that found no effect on fidelity was a high-quality, quantitative non-experimental study with a relatively large and representative sample and so these null findings should not be dismissed.

#### Audit and Provide Feedback

Four out of five studies (80%) testing ‘audit and provide feedback’ reported positive findings. Three quantitative/mixed methods studies found the strategy had a positive effect on fidelity. One qualitative study reported positive findings for program adoption. One quantitative study found no effect of ‘audit and provide feedback’ combined with training and coaching on fidelity.

#### Inform Local Opinion Leaders

Three qualitative studies (100%) assessing ‘inform local opinion leaders’ found the strategy to be positively associated with adoption. In these studies, the local opinion leaders were members of the school’s leadership team. The authors postulated that the school leaders supported program adoption by promoting the program and motivating the staff, allocating resources and planning implementation (Freeman et al., 2014; Lohrmann et al., 2008).

#### Improve Implementers’ Buy-in

Three qualitative studies (100%) reported positive findings for ‘improve implementers’ buy-in’. One study reported positive findings for fidelity. Two studies reported positive findings for adoption.

#### Use Train-the-Trainer Strategies

Two out of three quantitative/mixed methods studies (67%) testing ‘use train-the-trainer strategies’ found the strategy to be associated with improvements in fidelity. The other mixed methods study found that fidelity did not reach the acceptable level of 80% when ‘train-the-trainer’ was used in combination with other strategies.

#### Increase Demand and Expectations for Implementation

‘Increase demand and expectations’ refers to efforts to engage teachers with a new program by educating them about the program and its benefits. Both qualitative studies testing ‘increase demand and expectations for implementation’ found this strategy was positively associated with adoption, because if teachers had clear expectations about the program and perceived it to be valuable for their students, there was less resistance to program adoption within the classroom. These findings should be interpreted with caution as they were rated as not adequately derived from the data in both studies.

#### Organize School Personnel Implementation Team Meetings

Both qualitative studies (100%) testing ‘organize school personnel implementation team meetings’ found this strategy to be positively associated with adoption. One study was high quality and the other was medium quality.

#### Identify and Prepare Champions

One of the two qualitative studies (50%) testing ‘identify and prepare champions’ reported positive findings. The study with positive findings identified that having a program champion motivated teachers to adopt the program by highlighting its theoretical benefits (e.g. reduced workload and increased student support), and providing evidence of positive effects from pilot studies (Leadbeater et al., 2012). In this study, the champion also absorbed some of the responsibility of preparing/implementing the program to reduce the burden on the teacher, a known adoption barrier (Leadbeater et al., 2012). The other study found no evidence to suggest having a program champion improved fidelity, as high-quality implementation schools did not have a school champion whereas unexpectedly, low-quality implementation schools did. It can be hypothesised that low-quality implementation schools faced greater implementation challenges such that there was a greater need for a champion compared to high-quality schools. Both these studies were considered to be high quality.

#### Distribute Educational Materials

Neither of the two quantitative/mixed methods studies testing ‘distribute educational materials’ found a positive effect on program fidelity. These studies were high quality, however used small sample sizes *n* = 39 (Livet et al., 2018) and *n* = 3 (Fallon et al., 2018) respectively. They were potentially underpowered to detect an effect.

#### Conduct Local Needs Assessment

One out of two quantitative studies (50%) testing ‘conduct local needs assessment’ found the strategy had a positive effect on fidelity when used in combination with coaching and local technical assistance. Both studies were of high quality.

#### Remind School Personnel

Two studies assessing ‘remind school personnel’ reported positive findings. One RCT found a positive effect of emailed prompts on fidelity (Fallon et al., 2018). This study used a case study design with a small sample (*n* = 3), limiting its generalisability. A qualitative study reported that reminders were perceived to improve program adoption (Lohrmann et al., 2008).

#### Other Strategies

A quantitative study testing ‘make training dynamic’ found that providing an online mode of training delivery did not have a positive effect on program fidelity beyond the positive effect of in-person training. The quantitative study testing ‘develop instruments to monitor and evaluate core components of the innovation/new practice’ found the strategy had a positive effect on fidelity. Furthermore, the quantitative study testing ‘facilitation/problem-solving’ found a positive effect on fidelity (Hagermoser Sanetti et al., 2018). ‘Facilitation/problem-solving’ involved teachers participating in a pre-implementation logistical planning meeting where they identified potential implementation challenges and developed solutions.

A qualitative study found ‘facilitate relay of intervention fidelity and student data to school personnel’ was associated with program adoption when used with other strategies because it motivated staff to use the program as they understood that change was possible (Lohrmann et al., 2008).

Another qualitative study reported that ‘develop local policy that supports implementation’, ‘tailor strategies’ and ‘peer assisted learning’ facilitated adoption. The authors postulated that developing school policy and implementation guidelines enabled school staff to have a shared vision for how the program was supposed to be delivered, leading to more cohesive adoption by the school as a whole (Freeman et al., 2014). The authors also hypothesised that ‘tailor strategies’ helped the school take ownership over the program, which facilitated program adoption. Lastly the authors speculated that ‘peer assisted learning’ motivated teachers to adopt the program by learning about the program’s success in other schools. There were some quality concerns with this study, in that findings were not perceived to be adequately derived from the data and as such findings should be interpreted with caution.

A qualitative study testing ‘conduct cyclical small tests of change (piloting or trialling the practice first)’ found this strategy facilitated adoption because positive findings from the pilot trial motivated teachers to adopt the program (Leadbeater et al., 2012). Lastly, a qualitative study identified that ‘develop academic partnerships’, ‘build partnerships to support implementation’ and ‘conduct local consensus discussions’ were perceived by school administrators to facilitate program adoption.

### What Implementation Outcomes Were Assessed?

The most commonly assessed implementation outcome was fidelity, which was measured in 16 studies (76%) and defined as adherence (i.e. the extent to which the program was delivered as intended) in 7 studies; dosage (i.e. the quantity of the program delivered) in 4 studies; and quality of delivery (i.e. how well the program was delivered) in 3 studies. Adoption (i.e. official decisions/efforts from the school to use the program) was assessed in 5 qualitative studies (24%).

## Discussion

The primary aim of this review was to identify effective strategies that improve implementation outcomes for school-based universal mental health prevention programs. Twenty-two strategies (of 75) were identified as effective at enhancing fidelity and/or adoption, which were the only implementation outcomes tested (of 8). Consistent with prior research (Moore et al., 2021), most of the identified strategies were in SISTER domains of train and educate stakeholders (*n* = 5), develop stakeholder interrelationships (*n* = 5), support educators (*n* = 4), use evaluative and iterative strategies (*n* = 4) and provide iterative assistance (3).

The most promising strategies for implementation were audit and provide feedback, engaging principals as local opinion leaders, improving teachers’ buy-in and organising regular school team program meetings when used in combination with other strategies such as peer-assisted learning and developing school policies to support implementation. This partially supported findings from previous trials of school-based physical health programs in which audit and provide feedback and engaging the school leadership team as local opinion leaders increased program adoption in combination with other strategies such as provide educational materials and training (Sutherland et al., 2021; Wolfenden et al., 2017). We found audit and provide feedback to be the most promising strategy, consistent with other systematic reviews which found a moderate to large effect of audit and provide feedback on the implementation of classroom behavioural programs (Fallon et al., 2015; Merle et al., 2022; Noell et al., 2014; Solomon et al., 2012).

Our findings that efforts to improve teachers’ program buy-in and regular staff meetings improved adoption appear to be relatively novel. We found one qualitative study in which these strategies were found to facilitate adoption of a school-based physical activity program (Cassar et al., 2020). However, we could not identify any other implementation trials in which improving teachers’ program buy-in or organising regular program meetings for school staff was tested. It makes sense that improving teachers’ buy-in would increase adoption as lack of teacher buy-in is a barrier to program adoption (Herlitz et al., 2020). Similarly, having school teams meet regularly to problem solve implementation challenges would reduce barriers to program adoption. Our relatively novel and positive findings for improving teachers’ buy-in and holding regular staff meetings advances the evidence base about what works to enhance program adoption in schools.

Prior research has identified training as a core strategy for enhancing implementation of school-based programs (Fixsen et al., 2005; Smith et al., 2018). For this reason, training is commonly in-built as a strategy in the delivery model for mental health prevention programs in the real world (Kusche, 2012; PAXIS Institute, 2020). It was unexpected that 55% of studies in this review examining the effect of ongoing training on fidelity (the extent to which the program was delivered as intended) found null results. It is possible that ongoing training is more useful for enhancing adoption than fidelity. This was supported by our findings that training was positively associated with adoption when used in combination with other strategies, mirroring the results of previous RCTs of school-based physical health prevention programs (Sutherland et al., 2020; Wolfenden et al., 2019).

Similarly, the proportion of studies reporting positive effects of coaching on fidelity was lower than expected. Coaching has been identified as the implementation strategy most likely to lead to good fidelity and adoption of evidence-based practices in the broader implementation literature (Louie et al., 2021; Snyder et al., 2015). Coaching has also been found to be effective at improving fidelity for school-based mental health prevention programs (Smith et al., 2018; Stormont et al., 2015). Subsequently, coaching has been built into programming supports for at-scale mental health prevention programs such as the PAX Good Behaviour Game (PAXIS Institute, 2020) and SW-PBIS (Center on Positive Behavioral Interventions & Supports, 2015; PAXIS Institute, 2020). Our mixed findings regarding the effects of coaching do not suggest that it is ineffective, but rather highlight pragmatic gaps in our understanding about how coaching can be used to support implementation.

The implementation planning strategy identified in Hagermoser Sanetti et al.’s (2018) study did not fit neatly into the SISTER framework. The strategy involved a consultant meeting with teachers to walk through a logistical plan for program implementation including cope-ahead planning for predicted barriers (Hagermoser Sanetti et al., 2018). The process fit somewhere between the ‘develop a detailed implementation plan’ and ‘facilitation/problem-solving’ strategies. We categorised the strategy as facilitation/problem-solving given the focus on cope-ahead planning to overcome barriers and the non-evaluative nature of the process.

The second aim of this study was to identify the implementation outcomes assessed in implementation research in the field of school-based mental health. Most studies measured program fidelity, which is unsurprising, given implementation scientists assume fidelity is predictive of program outcomes (Berkel et al., 2011; Durlak & DuPre, 2008). From this review, it appears that program adoption is a relatively neglected area of implementation research in school-based mental health programs, which may reflect why few evidence-based programs are being adopted in the real world (Kretlow & Helf, 2013).

Surprisingly, the few studies that measured adoption found positive findings for the implementation strategy. This contradicted findings which suggested that existing evidence-based programs were not being successfully adopted in schools (Kretlow & Helf, 2013). Given the breadth of literature on the implementation challenges interfering with the adoption of evidence-based programs, it is possible this unexpected finding was due to a publication bias, in that only reports of strategies that successfully enhanced adoption were published. It is also possible that, since Kretlow and Helf’s (2013) finding is 9 years old, effective implementation strategies have since been developed and that our finding reflects the true state of affairs. This is plausible given that 86% of our studies assessing adoption were published between 2013 and 2020.

### Research Implications

This study suggests a number of implications for future research. First, the present review provides empirical support for the utility of some of the 75 implementation strategies considered by educational experts to be useful for enhancing school-based program implementation in the SISTER framework (Cook et al., 2019). We identified 22 SISTER-defined strategies that work to enhance program fidelity and adoption and 2 that do not appear to work but warrant further investigation. The next step is for researchers to identify the mechanisms, pre-conditions, moderators, and mediators which explain these effects. This will allow implementation scientists to build causal pathways models between implementation strategies and effectiveness outcomes for the SISTER implementation strategies (Lewis et al., 2018) to guide school-based implementation practice. Second, this study highlights that there are 51 SISTER-defined strategies that have yet to be studied in school-based mental health prevention research. Consistent with Moore et al.’s (2021) study, few if any studies reported strategies from SISTER domains of engage consumers, adapt and tailor to context, change infrastructure and use financial strategies. Future research should develop and trial strategies from these under-researched SISTER domains. Research into the SISTER-defined strategies is in its infancy given the framework was published only 3 years ago, so it is possible that practitioners are utilising these other strategies but have yet to empirically test them. Third, the findings show that existing research on school-based implementation strategies has been limited by case-study designs and small sample sizes. It is recommended future studies use larger samples and randomised controlled trials to test strategies against usual delivery controls to better understand their effect on implementation outcomes. Fourth, our study highlights the need for research focused on implementation strategies to enhance the sustainability of school-based universal mental health interventions so that these programs can yield long-term benefits for children and young adolescents.

### Practical Implications

There are five practical implications of our research. First, we recommend school staff select and tailor implementation strategies with positive findings from this review. This includes audit and provide feedback, engage the leadership team as local opinion leaders, improve teachers’ buy-in and organise school personnel meetings. We provide examples of what successful implementation of these strategies looks likes in practice in Supplementary File 4. Most notably, audit and provide feedback appears to work if school/project staff observe program implementation and provide teachers with positive reinforcement and practical tips for improvement (Fallon et al., 2018; Oliver et al., 2015; von der Embse et al., 2019). Engaging the leadership team as local opinion leaders appears to work if the project staff organise pre-buy-in meetings (Freeman et al., 2014; Hudson et al., 2020) and regular check-ins with principals and program leaders (Lohrmann et al., 2008). School personnel implementation team meetings appear to be time efficient when integrated into regular weekly staff meetings (Freeman et al., 2014; Hudson et al., 2020).

A second implication of our research is we recommend that schools plan and prepare for implementation by determining how to apply these strategies to the delivery model of programs for which they are not already in-built (which is most). For example, staff could develop an internal system for monitoring the program’s progress and providing feedback to instructors/program deliverers as a motivational tool. Third, it is recommended that program developers design new/refine existing programs to incorporate these implementation strategies into delivery models. Fourth, when tailoring of programs is needed for individual schools, it is recommended that researchers partner with school staff and conduct formative evaluations to identify which strategies are perceived by staff to be the most acceptable and feasible in their local context as mutually chosen strategies are most likely to be adopted and implemented with high fidelity. Fifth, it is recommended that strategies be iteratively adapted such that they continue to be relevant and effective over time.

### Strengths and Limitations

There were a number of strengths in this study. Databases from the fields of medicine, psychology, nursing and education were searched to capture the breadth of published literature in this multidisciplinary field of research. A high interrater reliability, as assessed by Cohen’s kappa, was obtained, which reduced the risk of bias at the study selection phase. Furthermore, risk of bias during analysis was further reduced by having two researchers perform a quality assessment rating for all studies. Finally, this study reviewed 67% of studies rated as high quality, which increases confidence in our findings. This proportion of high-quality studies is relatively large in the field of implementation science where pragmatic trials are often prioritised over traditional, more robust research trials (Wolfenden et al., 2017, 2021). Our adherence to Proctor et al.’s (2013) reporting guidelines increased the rigour and replicability of our findings, something that is lacking in research on mental health implementation strategies, in which temporality, justifications and outcomes are rarely reported (Hooley et al., 2020).

Our study differed to other systematic reviews examining the implementation of prevention programs in that we included qualitative and mixed methods studies (Rabin et al., 2010; Wolfenden et al., 2017, 2020) and focused only on school-based programs addressing mental health. These foci distinguish this review from prior reviews and are strengths as they enable a deeper understanding of the successful strategies specifically for mental health programs in schools. However, we acknowledge that the inclusion of qualitative studies may have reduced the rigour of our findings.

This is the first known study to apply the SISTER framework to a review of school-based implementation strategies. This is an important extension of this field as the SISTER framework overcomes issues with inconsistent strategy nomenclatures, which has limited findings of previous systematic reviews of prevention program implementation (Rabin et al., 2010; Wolfenden et al., 2017, 2020). Using SISTER as a categorical tool increased the specificity of our search terms. However, this might have been at the expense of sensitivity. It is possible our searches missed relevant studies in which implementation strategies were categorised in unsystematic terms which is common in the field of school-based implementation research.

Our study is distinguished from other systematic reviews of implementation strategies for mental health programs, which considered any type of program (prevention/clinical) delivered in any setting (hospital, clinical or school) (Novins et al., 2013; Powell et al., 2014). This is important given the growing mental health burden and demand for curriculum-based prevention programs in schools (UNICEF, 2021; World Health Organization, 2021).

There were also some study limitations to consider. Only articles published in English were included (including studies from high-income countries, i.e. 90% from the USA), which may skew the results and limit generalisability of findings more broadly. Given that lack of resources is a known barrier to school-based program implementation (Herlitz et al., 2020), it is possible that learnings from this study are not relevant to schools in low- and middle-income countries. There are also concerns that excluding non-English-language articles could create a language bias wherein program effectiveness is overestimated as English-language journals are more likely to publish positive findings (Grégoire et al., 1995). Two meta-analyses found minimal effects of language restrictions on program effects (Morrison et al., 2012; Nussbaumer-Streit et al., 2020). This increased our confidence that our systematic review was not affected by an English-language bias.

It is possible that our exclusion of grey literature studies could have introduced publication bias (Adams et al., 2016; McClain et al., 2021). A recent systematic review found that the exclusion of grey literature study data led to an overinflated effect size in a minority of meta-analyses; however, for most meta-analyses, there was no effect (Schmucker et al., 2017). This increased our confidence that our decision to exclude grey literature to control the quality of studies most likely did not lead to an overestimation of implementation strategy effects.

Our decision to include studies with self-report data could be considered a limitation, as self-report data has inherent risks of performance bias and demand characteristics. However, self-report data appears to be the most common type of data in implementation research (Last et al., 2021). Two systematic reviews of the implementation of prevention programs contained self-report data in 74% (Wolfenden et al., 2017) and 73% (Powell et al., 2014) of included studies respectively. We did not think it would be feasible to gain an accurate understanding of which strategies worked in practice to enhance implementation of prevention programs by excluding self-reported data.

Our decision not to exclude studies due to poor study design could be a limitation that undermined the rigour of our findings. However, we believe a strength of this decision was that it allowed us to more accurately document the state of the evidence base on implementation strategies in the school-based prevention program literature.

Our decision not to dual screen all title/abstracts and full texts could have undermined the reliability of our screening process. However, resources prohibited dual screening all records, which is common in systematic reviews and as such dual screening a random selection appears to be an acceptable solution (Page et al., 2021). We also only moved on to the next stage of the screening process once a high degree of inter-rater agreement was established (of 90% or higher), increasing confidence that we were able to correctly identify relevant publications even without full dual screening.

The included studies in this review had much smaller sample sizes compared to other systematic reviews of the implementation of prevention programs (Novins et al., 2013; Rabin et al., 2010; Wolfenden et al., 2017). Small sample sizes limited the generalisability of the quantitative findings identified in this review. Another limitation was that 67% of studies had no theoretical underpinning for the implementation strategy. This appears to be lower than other systematic reviews of implementation strategies for prevention programs; for example, Wolfenden et al. (2017) only identified 26% of their included studies to have no theoretical underpinning (Wolfenden et al., 2017). Strategies informed by evidence-based logic models/theories of change are more likely to be effective (Baffsky et al., 2021), and as such we recommend practitioners consider/utilise evidence-based logic models to guide strategy implementation.

In this review, we have identified qualitative studies that reported positive findings for engaging principals as local opinion leaders, increasing demand and expectations for implementation and organising school personnel implementation team meetings. The effectiveness of these strategies needs to be confirmed by future RCTs. The studies that assessed adoption were qualitative limiting what is known about the impact of implementation strategies on adoption in quantitative terms. Further quantitative research is needed to establish a criterion for acceptable adoption within schools and then examine how implementation strategies can be used to achieve this.

Future research should also compare how different strategies (discrete or multicomponent) affect different implementation outcomes. Existing systematic reviews have focused on the effects of strategies on fidelity (Fallon et al., 2015; Merle et al., 2022; Noell et al., 2014; Solomon et al., 2012; Stormont et al., 2015), limiting what is known about how strategies can be used to enhance other implementation outcomes such as adoption or sustainability in schools. Our review also identified that the accuracy of findings in school-based implementation research can be undermined by a high attrition due to staff turnover. Researchers should consider how trials can be structured to minimise risk of attrition due to staff contracts ending. For example, for a 1-year trial researchers should aim to collect baseline data at the start of the school year and final follow-up data at the end of the same school year.

## Conclusion

This review identified twenty-two implementation strategies that appear to work to enhance the fidelity or adoption of mental health prevention programs in schools. Strategies that showed the most promise included the monitoring and provision of feedback, engaging principals as program leaders, improving teachers’ buy-in and organising school personnel implementation meetings. Further research with practitioners and school staff is needed to identify which bundle of implementation strategies work best for whom and under what conditions. This would be ideally conducted using prospective RCTs with large samples and long-term follow-ups to create an evidence base for what works to improve the sustainability of mental health prevention programs in schools. As a practical consideration, given the time lag in this proposed translational research, we recommend school-based practitioners and researchers start trialling strategies found to be effective in this review as part of their continuous quality improvement.

## Supplementary Information

Below is the link to the electronic supplementary material.

Supplementary file1 (DOCX 21 KB)

Supplementary file2 (DOCX 32 KB)

Supplementary file3 (XLSX 27 KB)

Supplementary file4 (DOCX 45 KB)

Supplementary file5 (DOCX 40 KB)

## Data Availability

All study materials are available from the research team upon request to the lead investigators.
