# Supplementary material for: Strategies for Enhancing the Implementation of Universal Mental Health Prevention Programs in Schools: A Systematic Review
Source: Prev Sci. 2022 Sep 13;24(2):337–52. doi: 10.1007/s11121-022-01434-9 (PMC9938015; doi:10.1007/s11121-022-01434-9)
Supplement: Supplementary file 1 — Supplementary file1 (DOCX 21 KB) [file 11121_2022_1434_MOESM1_ESM.docx]

**Supplementary File 1: Search Strategies**

**PubMed:**

| #1 | "Mental Health"[MeSH Terms] OR "Mental Health"[Text Word] OR "emotional difficulties"[All Fields] OR "behavioural difficulties"[Text Word] OR "behavioral difficulties"[Text Word] OR "social-emotional"[Text Word] OR "Social Emotional Learning"[Text Word] OR "behaviour problems"[Text Word] OR behavioural[Title/Abstract] OR behavior*[Title/Abstract] OR "behavior problems"[Title/Abstract] OR "behavioural problems"[Title/Abstract] OR "behavioral problems"[Title/Abstract] OR "pro-social behaviour"[Title/Abstract] OR "pro-social behavior"[Title/Abstract] OR "disruptive behaviour"[Title/Abstract] OR "disruptive behavior"[Title/Abstract] |
| --- | --- |
| #2 | "schools*"[MeSH Terms] OR "school*"[Text Word] OR "teacher*"[Text Word] OR "classroom*"[Text Word] OR "pupil*"[Text Word] OR "middle school"[Text Word] OR "elementary school"[Text Word] OR "primary school"[Text Word] OR "high school"[Text Word] OR "school-based"[Text Word] OR “student”[Text Word] |
| #3 | "Evidence-Based Practice"[MeSH Terms] OR "Mental Health Services"[MeSH Terms] OR "intervention*"[Text Word] OR "program*"[Text Word] OR "initiative"[Text Word] OR "prevention"[Text Word] OR "evidence based practice*"[Text Word] OR "evidence based practice*"[Text Word] OR "EBP"[All Fields] OR "EBPs"[All Fields] OR "EBPP"[All Fields] |
| #4 | "Implementation Science"[MeSH Terms] OR "implementation"[Title/Abstract] OR "sustainability"[Title/Abstract] OR "acceptability*"[Title/Abstract] OR "fidelity"[Title/Abstract] OR "barriers and facilitators"[Title/Abstract] OR "adherence"[Title/Abstract] OR "adoption"[Title/Abstract] OR "integration*"[Title/Abstract] |
| #5 | NOT "college students"[All Fields]) NOT "undergraduate students"[All Fields]) NOT "medical students"[All Fields]) NOT "psychology students"[All Fields] |
| #6 | NOT “hospital” NOT “primary health care” NOT “primary care” NOT hospital-based OR “general practitioner” NOT nurse NOT “family medicine” NOT “general medicine” NOT nurse NOT clinician |
| #7 | Not “study protocol” |
| #7 | #1 AND #2 AND #3 AND #4 AND #5 AND #6 AND #7 |

Limit results to 2000-2021, English and Journal articles.

**PsycINFO:**

| **Concept** | **Key words and MESH terms** |
| --- | --- |
| Mental Health | SU.EXACT(("mental health" OR "mental healthcare")) OR SU.EXACT("Public Mental Health") OR "behavior problems" OR SU.EXACT("externalizing symptoms") OR AB("mental health") OR "behavioural problems" OR "behaviour problems" OR AB("social-emotional") OR SU.EXACT("Social Emotional Learning") OR "behavioral difficulties" OR "behavioral problems" OR AB(behaviour*) |
| School | AB(school*) OR SU.EXACT(teachers) OR SU.EXACT(Students) OR (teacher*) OR AB(classroom*) OR AB(pupil*) OR SU.EXACT("Primary Education") OR SU.EXACT(Secondary Education) OR SU.EXACT (Schools) OR SU.EXACT (Elementary Schools) OR SU.EXACT(High Schools) OR SU.EXACT(Junior High Schools) OR SU.EXACT(Middle Schools) OR “school-based” OR student |
| Intervention | SU.EXACT("Early Intervention") OR SU.EXACT("School Based Intervention") OR SU.EXACT("Preventative Mental Health Services") OR MAINSUBJECT.EXACT("Mental Health Programs") OR SU.EXACT(Primary Mental Health Intervention) OR MAINSUBJECT.EXACT("Evidence Based Practice") OR AB(intervention*) OR AB(program*) OR "evidence-based practice*" OR "evidence based practice*" OR EBP OR EBPs OR EBPP OR MAINSUBJECT.EXACT("Health Promotion") OR "evidence-based school programs" |
| Implementation | implementation OR "Implementation Science" OR uptake OR sustainability OR sustainment OR “barriers and facilitators” OR fidelity OR adoption OR acceptability |
| NOT | NOT noft("college students" OR "undergraduate students" OR "medical students" OR "psychology students" OR "higher education")) |
| NOT | “hospital” OR “primary health care” OR “primary care” OR hospital-based OR “general practitioner” OR nurse OR “family medicine” OR “general medicine” OR nurse OR clinician |

Limit results to 2000-2021, English and Journal articles.

**ERIC**

| **Concept** | **Key words and MESH terms** |
| --- | --- |
| Mental Health | SU.EXACT(("mental health" OR "mental healthcare")) OR SU.EXACT("Public Mental Health") OR SU.EXACT("behavioral disorder") OR SU.EXACT("behavior problems") OR SU.EXACT("externalizing symptoms") OR "mental*" OR AB("behavioural disorder*") OR "behaviour problems" OR AB("social-emotional") OR SU.EXACT("Social Emotional Learning") OR "behavioral difficulties" OR "behavioral problems" OR "disruptive behaviour" OR "disruptive behavior" |
| School | AB(school*) OR SU.EXACT(teachers) OR SU.EXACT(Students) OR (teacher*) OR (student*) OR AB(classroom*) OR AB(pupil*) OR SU.EXACT("Primary Education") OR SU.EXACT(Secondary Education) OR SU.EXACT (Schools) OR SU.EXACT (Elementary Schools) OR SU.EXACT(High Schools) OR SU.EXACT(Junior High Schools) OR SU.EXACT(Middle Schools) OR AB(student*) |
| Intervention | SU.EXACT("Early Intervention") OR SU.EXACT("School Based Intervention") OR SU.EXACT("Preventative Mental Health Services") OR MAINSUBJECT.EXACT("Mental Health Program Evaluation") OR MAINSUBJECT.EXACT("Mental Health Programs") OR SU.EXACT(Primary Mental Health Intervention) OR MAINSUBJECT.EXACT("Evidence Based Practice") OR AB(intervention*) OR AB(program*) OR "evidence-based practice*" OR "evidence based practice*" OR EBP OR EBPs OR EBPP OR MAINSUBJECT.EXACT("Health Promotion") OR "evidence-based school programs" |
| Implementation | implementation OR "Implementation Science" OR uptake OR AB(sustainability) OR sustainment OR barriers OR facilitators OR fidelity |

Limits: English, Peer-reviewed, Humans, 2000-2020, Education level (exclude preschool education, early childhood education, higher education, postsecondary education and adult education)

**CINAHL**

| **Concept** | **Key words and MESH terms** |
| --- | --- |
| Mental Health | (MH "Mental Health") OR (MH "Mental Health Promotion") OR "behavioral disorder" OR "behavior problems" OR “externalizing symptoms" OR "mental health" OR "behavioural disorder*" OR "behaviour problems" OR "social-emotional" OR "Social Emotional Learning" OR "behavioral difficulties" OR "behavioral problems" OR "disruptive behaviour" OR "disruptive behavior" OR “pro-social behaviour” OR “pro-social behavior” |
| School | (MH "School Mental Health Services") OR school* OR (MH "Students, High School") OR (MH "Students, Elementary") OR (MH "Students, Middle School") OR “student” |
| Intervention | "program" OR intervention OR initiative OR "evidence-based practice*" OR EBP OR EBPs OR “evidence-based school programs” or prevention |
| Implementation | (MH "Program Implementation") or (MH"Implementation Science") OR "implementation" OR adoption OR acceptability OR sustainability OR sustainment OR “barriers and facilitators” OR fidelity |
| NOT | (MM "Students, Medical") OR (MM "College and Universities") OR "college students" OR "undergraduate students" OR "medical students" OR "psychology students" OR "higher education” |
| NOT | primary health care or primary care or primary care nursing or family medicine or family practice or general medicine |
| NOT | Study protocol |

Limits: English, 2000-2021, Peer-reviewed, journals (no books)
