# Supplementary material for: Strategies for Enhancing the Implementation of Universal Mental Health Prevention Programs in Schools: A Systematic Review
Source: Prev Sci. 2022 Sep 13;24(2):337–52. doi: 10.1007/s11121-022-01434-9 (PMC9938015; doi:10.1007/s11121-022-01434-9)
Supplement: Supplementary file 4 — Supplementary file4 (DOCX 45 KB) [file 11121_2022_1434_MOESM4_ESM.docx]

Supplementary File 4: A full overview of the implementation strategies evaluated in included studies and their effect on implementation outcomes

| *First Author, Country, EBP* | *SISTER Strategies tested* | *Actor/Action/target* | *Temporality/dose* | *Justification* | *Implementation outcome/findings* |
| --- | --- | --- | --- | --- | --- |
| *Mixed Methods Studies* | | | | | |
| Poduska, 2014  USA  PAX GBG | - Conduct ongoing training - Provide ongoing consultation/coaching | *Actor(s)*: Coaches who are experts in program implementation  *Action*: Provides initial training followed by booster training. Conducts classroom observations of teachers’ program implementation across multiple coaching cycles.  *Target:* Teachers  *Determinants targeted:* Knowledge and beliefs about the intervention; self-efficacy; external change agents. | *Preparation*: Two days of initial training during preparation  *Implementation*: One day booster session 6 months post-baseline; three coaching cycles includes four biweekly visits of 90 minute duration. | Bandura’s Social Cognitive Theory | *Implementation outcome:* Fidelity  *Findings*: A greater proportion of teachers reported high fidelity program implementation at the beginning of coaching cycle 2 and 3 (65.4%) compared to at the end of coaching cycle 1 (0.4%). However, this did not reach the criterion of acceptable fidelity (80%), providing insufficient evidence that coaching works in combination with training. |
| Anyon, 2016  USA  Other | - Use train-the-trainer strategies - Conduct ongoing training - Provide ongoing consultation/coaching | *Actor(s):* Program creators, implementation team consisting of researchers, school behavioural specialists and social work interns  *Action*: Program creators train implementation team in delivery of evidence-based program; implementation team facilitates professional development, booster sessions and regular on-site and online support.   *Target*: Implementation team and school staff (trickle down)  *Determinants targeted*: Knowledge and beliefs about the intervention; self-efficacy. | *Preparation*: Two weeks of training the trainer. Followed by two days of initial training for school staff.  *Implementation*: Five ‘booster sessions’, one every two months; consultation/coaching provided twice a month. | Durlak and Dupre’s (2008) framework of effective implementation | *Implementation outcome:* Fidelity  *Findings*: School staff identified the initial training, ongoing consultation/coaching they received as important supports for high fidelity program implementation. However, fidelity did not reach the acceptable criterion of 80%, as such there was insufficient evidence that these strategies worked. |
| Livet, 2018  USA  Other | - Distribute educational materials - Conduct ongoing training - Provide local technical assistance - Audit and provide feedback | *Actor(s):* Program purveyors and creators  *Action*: Provide multimedia, online training with assessment and accreditation. Supplement with downloadable resources such as manuals and activity worksheets. Offer online technical assistance (TA) to ‘ask-an-expert’ for practical tips in real-time and participate in Q&A. Provide real-time adherence (fidelity) tracking mechanism with student progress feedback loop.  *Target:* School staff  *Determinants targeted:* Knowledge and beliefs about the intervention; self-efficacy; external change agents; reflecting and evaluating. | *Preparation*: Online training and educational resources accessible as needed.  *Implementation*: Live TA and feedback loops provided weekly; Additional TA provided in a frequency/amount as needed. | Evidence-Based System for Innovation  Support model | *Implementation outcome*: Fidelity  *Findings*: There was evidence that training dosage, technical assistance initiation, speed in which technical assistance request was resolved and providers’ use of student progress reports for performance monitoring significantly predicted high provider adherence (fidelity) which in turn predicted students’ socioemotional growth. Access to educational resources was not a significant predictor of program fidelity. |
| *Quantitative Randomised Controlled Trials* | | | | | |
| Becker, 2014  USA  PAX GBG + PATHS | - Conduct ongoing training - Make training dynamic - Provide ongoing consultation/coaching | *Actor(s):* Program creators and coaches  *Action:* Provide either online or in-person training. This involves 12 online, multimedia modules with instruction activities on key program elements. Online training differs to in-person training as it does not include group discussions or lesson planning. Training followed by in-person coaching involving program material preparation, modelling, observation, feedback and practical support.  *Target*: Teachers  *Determinants targeted*: Knowledge and beliefs about the intervention; self-efficacy; external change agents. | *Preparation*: Training held 1-week before implementation. Online training duration was 5-8 hours and self-paced. In-person training was 8 hours.  *Implementation*: Coaching was provided as needed for 31 weeks during implementation (average 7.81 hours of coaching across this period). | Research suggesting that that online training could be as effective as in-person training at improving implementation of school-based programs (Bishop et al., 2005; Pianta et al. 2008). | *Implementation outcome*: Fidelity  *Findings*: Program fidelity was comparably high for teachers who received in-person versus online training. This suggests both modes of training had a positive effect on fidelity when combined with ongoing consultation/coaching.  No effect was found for making training dynamic by using the online mode of delivery. |
| Bradshaw, 2008  USA  SW-PBIS | - Conduct ongoing training - Provide ongoing consultation/coaching | *Actor(s):* Program creators and coaches  *Action*: Provide initial training with instructions on key program elements. Revise content through booster training sessions. Coaches provide ongoing support to translate lessons from training into practice.  *Target*: School staff (principals, teachers, support staff)  *Determinants targeted:* Knowledge and beliefs about the intervention; self-efficacy; external change agents. | *Preparation*: 2 day initial training.  *Implementation*: 1 day booster training per year; coaching supports four times per year for a period of 3 years. | Evidence that training in SW-PBIS had a positive effect on program adoption and sustainment (Nersesian et al. 2000; Taylor-Greene & Kartub, 2000) | *Implementation outcome*: Fidelity  *Findings*: Trained schools implemented the SW-PBIS intervention with higher fidelity at 1 and 2 year time points compared to untrained schools. Untrained schools increased in mean fidelity scores from 42% to 60% whereas trained schools increased from 36% to 88%, with the latter reaching acceptable fidelity. As such, training and coaching worked to improve fidelity. |
| Bradshaw, 2010  USA  SW-PBIS | - Conduct ongoing training - Provide ongoing consultation/coaching - Use train-the-trainer strategies | *Actor(s):* Program creators and coaches  *Action*: Initial training focused on reviewing core program elements and developing an implementation plan. Program leaders in schools trained to deliver professional development to other staff. External coaches provide ongoing implementation support, however type and quantity was unclear to the researchers.  *Target*: School staff (principals, teachers, support staff)  *Determinants targeted*: Knowledge and beliefs about the intervention; self-efficacy; planning; executing; access to knowledge and information; leadership engagement; external change agents. | *Preparation*: 2 day initial training for program leaders. Leaders deliver additional 2 days of professional development for other staff.  *Implementation*: 2 days of booster training per year; ongoing coaching for a period of 5 years. | Evidence training in SW-PBIS had a positive effect on program adoption and sustainment (Barrett et al., 2008; Nersesian et al., 2000) | *Implementation outcome*: Fidelity  *Findings:* Trained schools implemented the SW-PBIS with higher fidelity at 1, 2, 3 and 4 year follow up compared to untrained schools. Untrained schools increased in mean fidelity scores from 42% to 46% between baseline and 4 year follow up whereas trained schools increased from 36% to 92% over this period. This provided evidence that training and coaching worked to improve fidelity. Developing an implementation plan appears to be useful within training. Also, a train-the-trainer approach is useful for sustainability, to ensure existing school staff can train new staff, to overcome the cost barrier of having to pay for new staff to be trained and losing program expertise due to staff turnover. |
| Reinke, 2012  USA  PAX GBG | - Provide ongoing consultation/coaching | *Actor*(s): External coaches who are experts in program delivery.  *Action*: Provide motivational interviewing, modelling, monitor and provide visual performance feedback, role-playing, action planning and collaborative problem solving.  *Target*: Teachers  *Determinants targeted*: Knowledge and beliefs about the intervention; self-efficacy; individual stage of change; planning; external change agents; reflecting and evaluating. | *Preparation and implementation*: Biweekly coaching support for a period of one year. | Research suggesting that motivational interviewing and visual performance feedback were associated with improved implementation (Frey et al., 2011; Reinke et al., 2011; Reinke et al., 2007) | *Implementation outcome*: Fidelity  *Findings*: Teachers who received standard coaching support had fidelity scores of 87% at baseline and 90% at 1 year follow up. This was comparable to teachers in the enhanced Classroom Check-up coaching support condition who had fidelity scores of 84% and 96% at baseline and follow up respectively. In sum, additional coaching did not have an effect on fidelity. |
| Fallon, 2018  USA  PAX GBG | - Conduct ongoing training - Distribute educational materials - Audit and provide feedback - Remind school personnel | *Actor(s):* Research team  *Action*: Provide brief one-on-one training involving verbal review of implementation steps, Q&A and distribution of written manual. Send emailed implementation prompts reminding staff of steps and providing sample dialogue to guide implementation. Send emailed performance feedback including a graph of the percentage of implementation steps successfully completed the previous day, praise and reminder of steps to improve on.  *Target*: Teachers  *Determinant targeted*: Knowledge and beliefs about the intervention; self-efficacy; planning; reflecting and evaluating. | *Preparation*: One-off training lasting 15 minutes.  *Implementation*: Daily email prompts for 1 week followed by daily emailed performance feedback for one week. | Research that providing emailed feedback and implementation prompts can enhance fidelity (Hemmeter et al,. 2011; Simonsen et al., 2013) | *Implementation outcome*: Fidelity  *Findings*: The mean program fidelity for the three teachers was 67% after their didactic training and then significantly increased to 82% after email prompts (acceptable=80%) and then further increased to 91% after receiving performance feedback. This suggested that training and distributing educational resources did not work, however sending email reminders to school personnel and providing feedback did work. |
| *Quantitative Non-randomised Studies* | | | | | |
| von der Embse, 2012  USA  SW-PBIS | - Conduct ongoing training - Provide ongoing consultation/coaching - Audit and provide feedback | *Actor(s):* Program creators, school psychologist, project staff  *Action:* Program creators provide multi-media interactive professional development workshop on trauma-informed classroom practices. School psychologist provide training on universal screening to detect at-risk students and refer out. Project staff acting as coaches observe evidence-based program delivery, graph results and provide feedback.  *Target:* Teachers  *Determinants targeted*: Knowledge and beliefs about the intervention; self-efficacy; external change agents; reflecting and evaluating. | *Preparation*: 2 hour trauma-informed training; 90-minute universal screening training.  *Implementation*: Weekly coaching and feedback for two year trial period. | Research that training combined with ongoing coaching is integral to effective program implementation (Fixsen et al., 2005) | *Implementation outcome*: Fidelity  *Findings*: Training was associated with improvements in program fidelity. Before the intervention, approximately 33% of teachers reached acceptable program fidelity (80%) averaged across the eight program domains, and this increased to 58% of teachers who reached acceptable fidelity after 1 year. Whilst this was a significant aggregate increase, there was not a sufficient proportion of teachers delivering consistent high fidelity programming for the implementation strategies to be considered effective. |
| Pas, 2015  USA  PAX GBG | - Provide ongoing consultation/coaching - Conduct local needs assessment - Provide local technical assistance | *Actor(s):* External coaches experienced in teaching and program implementation.  *Action*: Provide modelling, needs assessment using observations, technical assistance involving feedback and support with action planning and check-ins.  *Target*: Teachers  *Determinants targeted*: Knowledge and beliefs about the intervention; self-efficacy; external change agents. | *Implementation*: At least one coach contact of a minimum 5 minutes per week for 31 weeks. Type, frequency and intensity of coaching varied according to teachers’ needs. | Research that coaching enhances implementation when combined with training (Wehby et al., 2012) | *Implementation outcome*: Fidelity  *Findings:* Teachers who received moderate and high amounts of coach contacts were more likely to implement the program more frequently (higher dosage) compared to teachers who received low amounts of coaching. This provides some evidence that coaching works. Needs assessments and technical assistance were provided under the umbrella of general coaching so it was hard to isolate them as being effective or ineffective. |
| Johnson, 2018  USA  PAX GBG | - Provide ongoing consultation/coaching - Conduct local needs assessment - Provide local technical assistance | *Actor(s):* External coaches experienced in teaching and program implementation.  *Action*: Provide four coaching activities; modelling, needs assessments using walk-through observations, technical assistance involving feedback and support with action planning and check-ins.  *Target*: Teachers  *Determinants targeted*: Knowledge and beliefs about the intervention; self-efficacy; external change agents. | *Implementation*: one coach contact of a minimum 5 minutes per week for 31 weeks. | Research suggesting coaching can be used to enhance program fidelity (Bradshaw et al. 2008; Kretlow & Bartholomew, 2010; Pas et al. 2014) | *Implementation outcome*: Fidelity  *Findings*: Modelling (as a coaching activity) was found to have a direct effect on program dosage. Conducting local needs assessment and providing technical assistance was not found to have an effect on program dosage or quality. |
| McDaniel, 2013  USA  SW-PBIS | - Conduct ongoing training - Provide ongoing consultation/coaching | *Actor(s):* External coaches experienced in teaching and program implementation.  *Action*: Provide initial training focused on setting expectations, action planning and developing reinforcement systems. Then provide ongoing telecoaching to review implementation components, monitor and provide feedback and problem solve implementation challenges.  *Target*: School staff (teachers, principals, support staff)  *Determinants targeted*: Knowledge and beliefs about the intervention; self-efficacy; external change agents. | *Preparation*: A one-off 2 day training workshop.  *Implementation*: Monthly coaching lasting 25-70 minutes each for a period of one year. | Research suggesting that coaching enhances fidelity when used in combination with training (Kretlow & Batholomew, 2010; Bradshaw et al., 2010) | *Implementation outcome*: Fidelity  *Findings*: Before training and telecoaching, none of the four schools had any SW-PBIS features in place (zero fidelity). Post-intervention, they all demonstrated high fidelity with scores ranging from 81-94% (over the threshold). This provided evidence that training and coaching worked to improve fidelity. |
| Oliver, 2015  USA  PAX GBG | - Provide ongoing training - Audit and provide feedback - Develop instruments to monitor and evaluate core components of the innovation/new practice | *Actor(s):* Researchers  *Action:* Provide interactive, multimedia training with instructions on program delivery and opportunities to practice. Observe program implementation and provided feedback through a) graph of implementation b) praising components delivered correctly c) correcting components to improve upon. Then distribute self-monitoring checklist listing 13 program components for teachers’ to hold themselves accountable to implement.  *Target*: Teachers  *Determinants targeted:* Knowledge and beliefs about the intervention; self-efficacy; external change agents; reflecting and evaluating. | *Preparation*: Initial training lasted 60-90 minutes.  *Implementation*: Audit and provide feedback was used for approximately 25 minutes a minimum of 5 times across 8-13 days (until teachers implemented all program components). Self-monitoring checklist used for 9-17 days. | Bandura’s Social Cognitive Theory | *Implementation outcome*: Fidelity  *Findings*: Program dosage was 0% for the four teachers at baseline and then increased to between 80-100% after receiving training and performance feedback. This high quantity implementation was sustained after the self-monitoring checklist was introduced. This provides evidence that ‘provide ongoing training’, ‘audit and provide feedback’ and ‘develop instruments to monitor and evaluate core components of the innovation/new practice’ work when used in combination. |
| Hagermoser  Sanetti, 2018  USA  SW-PBIS | - Facilitation/problem- solving - Provide ongoing consultation/coaching | *Actor(s):* Researchers acting as coaches  *Action*: Conduct an interview to identify classroom management challenges. Observe and document existing classroom practices. Use data to recommend new practices. Collaboratively develop an implementation action plan listing steps for new practices, identifying logistical barriers and cope-ahead strategies. Model challenging steps, observe the teacher practicing these steps and provide feedback.  *Target*: Teachers  *Determinants targeted*: Knowledge and beliefs about the intervention; self-efficacy; external change agents. | *Preparation:* One-off interview; observations 2-3 times per week.  *Implementation*: Observations 2-3 times per week; implementation planning occurred as needed when teachers scored less than 80% program adherence on two consecutive days. Modelling was provided when teachers’ adherence was less than 70% after implementation planning phase. | Positive results from a pilot study showing coaching involving implementation planning improved program fidelity (Sanetti & Collier-Meek, 2015) | *Implementation outcome:* Fidelity  *Findings*: Implementation planning (facilitation/problem solving) consistently increased teachers’ program quality such that they achieved quality of 80% or greater post-implementation planning. Teachers’ program adherence increased to a weak-moderate extent when they received participating modelling (a form of coaching) after implementation planning. Coaching did not have a positive effect on program quality. This suggests facilitation/problem solving increases program quality and coaching increases adherence. |
| Cook, 2015  USA  SW-PBIS | - Conduct ongoing training - Use train-the-trainer strategies - Improve implementers’ buy-in - Provide ongoing consultation/coaching | *Agent(s):* Researchers and external coaches with experience working in schools  *Action*: Provide ongoing interactive training to school implementation team, building their capacity to internally train other staff. Training focused on enhancing beliefs, attitudes, knowledge and skills to increase program fidelity. Provide coaching in between training sessions to support implementation.  *Target*: School staff (principals, teachers, support staff)  *Determinants targeted*: Knowledge and beliefs about the intervention; self-efficacy; individual stage of change; planning; external change agents. | *Implementation*: Each training day was 6 hours. There were 8 training days in year one, 6 in year two and 4 in year three. | Theory of planned behaviour | *Implementation outcome:* Fidelity  *Findings*: The supportive belief intervention successfully improved beliefs towards the implementation of the SW-PBIS, which was associated with improvements in program fidelity. This suggests train-the trainer, ongoing training and coaching strategies focused on self-efficacy and staff buy-in are effective at enhancing implementation. |
| *Qualitative Studies* | | | | | |
| Lohrmann, 2008  USA  SW-PBIS | - Provide local technical assistance - Inform local opinion leaders - Improve implementers’ buy-in - Increase demand and expectations for implementation - Facilitate relay of intervention fidelity and student data to school personnel - Provide ongoing consultation/coaching - Conduct ongoing training - Remind school personnel | *Actor(s):* External educational consultants  *Actions:* Provide support to the leadership team involving a pre-buy in meeting to establish rapport and expectations; regular check-ins and updates and coaching to resolve implementation challenges. Improve staff’s buy-in and expectations by presenting a logical rationale, evidence of effectiveness in similar contexts and school data highlighting need for program. Use pilot data and teacher testimonials to show staff success of early program efforts. Provide ongoing training and regular reminders with implementation instructions.  *Target*: School staff (principals, teachers, support staff)  *Determinants targeted*: Knowledge and beliefs about the intervention; self-efficacy; individual stage of change; planning; external change agents; leadership engagement. | *Preparation*: Pre-buy-in meeting with leadership team, initial training.  *Implementation*: Local technical assistance, leadership team check ins, consultations to improve staff buy-in, relay of intervention data to school staff, reminders to school staff as needed. | Research suggesting that leadership support, coaching, data as an engagement tool, training and reminders were facilitators of program adoption (Kincaird et al., 2007) | *Implementation outcome*: Adoption  *Findings*: Educational consultants perceived eight strategies to be effective at overcoming staff’s resistance to program adoption within schools. These strategies included consultants providing technical assistance and coaching to school leadership team to support implementation activities; improving staff’s program buy-in by providing them with evidence of the program being effective in other contexts (used success stories); where possible relaying data about the implementation of the intervention to teachers as an engagement tool and using training and reminders to build skills and knowledge. |
| Freeman, 2014  Australia  Other | - Audit and provide feedback - Develop local policy that supports implementation - Increase demand and expectations for implementation - Develop educational materials - Inform local opinion leaders - Adapt and tailor to context - Conduct ongoing training - Peer assisted learning - Organize school personnel implementation team meetings | *Actor(s):* School-based implementation team; researchers  *Action(s):* Provide training focused on program content, skills-building and role-playing. Use school data to increase staff’s awareness to need for program. Encourage leadership team to set expectations and promote program to other staff. Develop policy for responding to incidents and program implementation guidelines. Adapt program curriculum to local student needs. Organise a peer learning day for principals of different schools to come together and present their experiences of program implementation. Dedicate time in regular staff meetings to discuss program implementation. Monitor progress using school-level data and provide reinforcing graphical feedback during professional development.  *Target:* School staff (principals, teachers, support staff)  *Determinants targeted:* knowledge and beliefs about the intervention; self-efficacy; individual stage of change; planning; external change agents; networks and communications; leadership engagement; available resources (training); relative priority; cosmopolitanism. | *Preparation*: Initial training; leadership team engagement; develop policies and implementation guidelines.  *Implementation*: Discuss implementation in staff meetings; adapt program as needed; additional training; monitor and provide feedback; peer learning day.  Dose not quantified due to qualitative nature of research. | Research on the facilitators of effective program implementation in school settings (Han & Weiss, 2005; Higgins et al., 2012) | *Implementation outcome*: Adoption  *Findings*: These nine strategies were identified as facilitators of program adoption in qualitative interviews that used the significant change stories technique. |
| Mendenhall, 2013  USA  Other | - Conduct ongoing training - Provide ongoing consultation/coaching | *Actor(s):* External consultants  *Action*: Provide instructions on program components, available resources, evaluation tools and opportunities to practice skills. Provide on-site consultations to motivate, plan and boost morale for ongoing implementation.  *Target:* School staff (principals, teachers, school staff)  *Determinants targeted*: Knowledge and beliefs about the intervention; self-efficacy; individual stage of change; planning; external change agents. | *Implementation*: Training and consultation combined.  Dose not quantified due to qualitative nature of research | Research suggesting lack of training and ongoing support are barriers for program adoption (Anderson-Butcher et al., 2010; Mellin & Weist, 2011). | *Implementation outcome*: Adoption  *Findings*: Forty-five percent of interviewees identified ongoing training as a facilitator of program adoption. Twenty-five percent of interviewees identified having ongoing consultation as a facilitator of successful program adoption. |
| Hudson, 2020  USA  Other | - Inform local opinion leaders - Organize school personnel implementation team meetings - Identify and prepare champions | *Actor(s):* School leadership team and researchers  *Action*: Engage principals as local opinion leaders by promoting the benefits of the program for students and staff. Encourage principals to organise program timetable. Discuss program implementation in regular staff meetings. Encourage self-selection of program champions to overcome adoption resistance among other staff.  *Target*: School staff (principals, teachers and support staff)  *Determinants targeted*: Leadership engagement; networks and communication; champions. | *Preparation*: Engage principals as local opinion leaders.  *Implementation*: Organize program discussion in weekly staff meeting. Identify champions.  Dose not quantified due to qualitative nature of research | Consolidated Framework for Implementation Research | *Implementation outcome:* Fidelity  *Findings:* Schools with high implementation quality (fidelity) were distinguished from those with low implementation quality based on a) the leadership team’s engagement with the program as local opinion leaders and b) having regular implementation team meetings. There was no evidence to suggest high and low implementation quality schools were distinguished by the presence of a program champion. |
| Leadbeater, 2012  USA  Other | - Improve implementers’ buy-in - Identify and prepare champions - Conduct cyclical small tests of change (piloting or trialling the practice first) | *Actor(s):* Self-selected program champions  *Action(s):* Self-selecting champions pilot new program in their own classroom and then shared the program benefits with other staff. Explain to staff that the program will reduce their workload by reducing negative incidents they have to respond to.  *Target:* School staff (principals, teachers, support staff)  *Determinants targeted:* Individual stage of change, networks and communication, champions. | *Preparation*: Champions self-select and pilot program, encourage others to adopt program.  Dose not quantified due to qualitative nature of research | Literature on knowledge transfer mechanisms (Kolko et al., 2010) and marketing strategies to promote dissemination (Wharf Higgins, 2011). | *Implementation outcome*: Adoption  *Findings*: Program champions reported a key strategy for motivating teachers to adopt the program was showing them how it would reduce their workload and support their students. Program champions stated they often took on the role of preparing/implementing the program to ease the burden to teachers. Champions also found that positive findings from pilot studies served to motivate teachers to adopt the program. |
| Arnold, 2020  USA  Other | - Develop academic partnerships - Build partnerships to support implementation - Conduct local consensus discussions | *Actor(s):* Researchers and leadership team  *Action:* Invite leadership team to participate in a research study explaining they will have access to additional program resources such as educational materials, incentives and support staff. Establish reputability by providing evidence of previous rigorous research. Build partnerships with external mental health clinicians to come in and deliver programming. Discuss decision to adopt program with school staff.  *Target:* School staff (principals, teachers and support staff)  *Determinants targeted*: Cosmopolitanism; external change agents; relative priority. | *Preparation*: Develop academic partnerships; partner with external mental health clinicians; conduct local consensus discussions.  Dose not quantified due to qualitative nature of research | Domitrovich’s (2008) social-ecological implementation model | *Implementation outcome:* Adoption  *Findings*: School administrators identified that partnerships with reputable universities, partnerships with external mental health clinicians and collaborative decision-making with staff facilitated their decision to adopt the program. |
