# Supplementary material for: Strategies for Enhancing the Implementation of Universal Mental Health Prevention Programs in Schools: A Systematic Review
Source: Prev Sci. 2022 Sep 13;24(2):337–52. doi: 10.1007/s11121-022-01434-9 (PMC9938015; doi:10.1007/s11121-022-01434-9)
Supplement: Supplementary file 5 — Supplementary file5 (DOCX 40 KB) [file 11121_2022_1434_MOESM5_ESM.docx]

**Supplementary file 5:** Frequency of SISTER-defined strategies evaluated in included studies

| **#** | **SISTER-defined strategy** | **Number and percentage of studies testing strategy** | **References** |
| --- | --- | --- | --- |
| 1 | Conduct ongoing training | 14 (67%) | (Anyon et al., 2016; Becker et al., 2014; Bradshaw et al., 2010; Bradshaw et al., 2008; Cook et al., 2015; Fallon et al., 2018; Freeman et al., 2014; Livet et al., 2018; Lohrmann et al., 2008; McDaniel & Bloomfield, 2020; Mendenhall et al., 2013; Oliver et al., 2015; Poduska & Kurki, 2014; von der Embse et al., 2019) |
| 2 | Provide ongoing consultation/coaching | 14 (67%) | (Anyon et al., 2016; Becker et al., 2014; Bradshaw et al., 2010; Bradshaw et al., 2008; Cook et al., 2015; Hagermoser Sanetti et al., 2018; Johnson et al., 2018; Lohrmann et al., 2008; McDaniel & Bloomfield, 2020; Mendenhall et al., 2013; Pas et al., 2015; Poduska & Kurki, 2014; Reinke et al., 2012; von der Embse et al., 2019) |
| 3 | Audit and provide feedback | 5 (24%) | (Fallon et al., 2018; Freeman et al., 2014; Livet et al., 2018; Oliver et al., 2015; von der Embse et al., 2019) |
| 4 | Provide local technical assistance | 4 (19%) | (Johnson et al., 2018; Livet et al., 2018; Lohrmann et al., 2008; Pas et al., 2015) |
| 5 | Inform local opinion leaders | 3 (14%) | (Freeman et al., 2014; Hudson et al., 2020; Lohrmann et al., 2008) |
| 6 | Improve implementers’ buy-in | 3 (14%) | (Cook et al., 2015; Leadbeater et al., 2012; Lohrmann et al., 2008) |
| 7 | Use train-the-trainer strategies | 3 (14%) | (Anyon et al., 2016; Bradshaw et al., 2010; Cook et al., 2015) |
| 8 | Increase demand and expectations for implementation | 2 (9%) | (Freeman et al., 2014; Lohrmann et al., 2008) |
| 9 | Organize school personnel implementation team meetings | 2 (9%) | (Freeman et al., 2014; Hudson et al., 2020) |
| 10 | Identify and prepare champions | 2 (9%) | (Hudson et al., 2020; Leadbeater et al., 2012) |
| 11 | Distribute educational materials | 2 (9%) | (Fallon et al., 2018; Livet et al., 2018) |
| 12 | Conduct local needs assessment | 2 (9%) | (Johnson et al., 2018; Pas et al., 2015) |
| 13 | Make training dynamic | 1 (5%) | (Becker et al., 2014) |
| 14 | Remind school personnel | 1 (5%) | (Fallon et al., 2018) |
| 15 | Develop instruments to monitor and evaluate core components of the innovation/new practice | 1 (5%) | (Oliver et al., 2015) |
| 16 | Facilitation/problem-solving | 1 (5%) | (Hagermoser Sanetti et al., 2018) |
| 17 | Facilitate relay of intervention fidelity and student data to school personnel | 1 (5%) | (Lohrmann et al., 2008) |
| 18 | Develop local policy that supports implementation | 1 (5%) | (Freeman et al., 2014) |
| 19 | Tailor strategies | 1 (5%) | (Freeman et al., 2014) |
| 20 | Peer assisted learning | 1 (5%) | (Freeman et al., 2014) |
| 21 | Conduct cyclical small tests of change (piloting or trialling the practice first) | 1 (5%) | (Leadbeater et al., 2012) |
| 22 | Develop academic partnerships | 1 (5%) | (Arnold et al., 2020) |
| 23 | Build partnerships to support implementation | 1 (5%) | (Arnold et al., 2020) |
| 24 | Conduct local consensus discussions | 1 (5%) | (Arnold et al., 2020) |
